# Inhibition of C3 with pegcetacoplan results in normalization of hemolysis markers in paroxysmal nocturnal hemoglobinuria

**DOI:** 10.1007/s00277-022-04903-x

**Published:** 2022-07-22

**Authors:** Raymond S. M. Wong, Humphrey W. H. Pullon, Ismail Amine, Andrija Bogdanovic, Pascal Deschatelets, Cedric G. Francois, Kalina Ignatova, Surapol Issaragrisil, Pimjai Niparuck, Tontanai Numbenjapon, Eloy Roman, Jameela Sathar, Raymond Xu, Mohammed Al-Adhami, Lisa Tan, Eric Tse, Federico V. Grossi

**Affiliations:** 1 Sir Y.K. Pao Centre for Cancer and Department of Medicine and Therapeutics, Prince of Wales Hospital, The Chinese University of Hong Kong, Shatin, Hong Kong, China; 2 Waikato Hospital, Hamilton, New Zealand; 3 Tokuda Hospital, Sofia, Bulgaria; 4 Clinic of Hematology, Clinical Center of Serbia, Faculty of Medicine, University of Belgrade, Belgrade, Serbia; 5 Apellis Pharmaceuticals, 100 5th Avenue, Waltham, MA 02451 USA; 6 National Specialized Hospital for Active Treatment of Hematologic Diseases, Sofia, Bulgaria; 7 Faculty of Medicine Siriraj Hospital, Bangkok, Thailand; 8 Ramathibodi Hospital, Bangkok, Thailand; 9 Phramongkutklao Hospital and Phramongkutklao College of Medicine, Bangkok, Thailand; 10 Lakes Research, Miami Lakes, FL USA; 11 Department of Hematology, Ampang Hospital, Selangor, Malaysia; 12 Lisa Tan Pharma Consulting Ltd, Cambridge, UK; 13 Department of Medicine, The University of Hong Kong, Pokfulam, Hong Kong, China

**Keywords:** Paroxysmal nocturnal hemoglobinuria (PNH), Phase I/II trials, Hemoglobin, LDH, Safety, Quality of life

## Abstract

Paroxysmal nocturnal hemoglobinuria (PNH) is a rare, acquired hematologic disorder characterized by complement-mediated hemolysis. C5 inhibitors (eculizumab/ravulizumab) control intravascular hemolysis but do not prevent residual extravascular hemolysis. The newly approved complement inhibitor, pegcetacoplan, inhibits C3, upstream of C5, and has the potential to improve control of complement-mediated hemolysis. The PADDOCK and PALOMINO clinical trials assessed the safety and efficacy of pegcetacoplan in complement inhibitor-naïve adults (≥ 18 years) diagnosed with PNH. Patients in PADDOCK (phase 1b open-label, pilot trial) received daily subcutaneous pegcetacoplan (cohort 1: 180 mg up to day 28 [*n* = 3]; cohort 2: 270–360 mg up to day 365 [*n* = 20]). PALOMINO (phase 2a, open-label trial) used the same dosing protocol as PADDOCK cohort 2 (*n* = 4). Primary endpoints in both trials were mean change from baseline in hemoglobin, lactate dehydrogenase, haptoglobin, and the number and severity of treatment-emergent adverse events. Mean baseline hemoglobin levels were below the lower limit of normal in both trials (PADDOCK: 8.38 g/dL; PALOMINO: 7.73 g/dL; normal range: 11.90–18.00 g/dL), increased to within normal range by day 85, and were sustained through day 365 (PADDOCK: 12.14 g/dL; PALOMINO: 13.00 g/dL). In PADDOCK, 3 serious adverse events (SAE) led to study drug discontinuation, 1 of which was deemed likely related to pegcetacoplan and 1 SAE, not deemed related to study drug, led to death. No SAE led to discontinuation/death in PALOMINO. Pegcetacoplan was generally well tolerated and improved hematological parameters by controlling hemolysis, while also improving other clinical PNH indicators in both trials. These trials were registered at www.clinicaltrials.gov (NCT02588833 and NCT03593200).

**Supplementary Information:**

The online version contains supplementary material available at 10.1007/s00277-022-04903-x.

## Introduction

Paroxysmal nocturnal hemoglobinuria (PNH) is a rare, acquired, life-threatening hematologic disease [1, 2]. This disease is characterized by complement-mediated hemolysis with or without hemoglobinuria, an increased susceptibility to thrombotic episodes, and/or some degree of bone marrow failure [3, 4]. The onset of the disease is often insidious [5], and although there have been reports of spontaneous remission, the course of the disease is generally chronic and progressive [6].

Complement-mediated lysis of red blood cell (RBC) clones, one of the primary clinical manifestations in PNH, is caused by the lack of functional GPI-linked proteins CD55 and CD59 on their surfaces to protect them against this process [1, 7–9]. This deficiency of key inhibitory regulators of the complement pathway at the RBC surface makes them particularly susceptible to the membrane attack complex (MAC), leading to lysis in the presence of complement activation [10, 11]. In PNH, uncontrolled complement activation leads to intravascular hemolysis (IVH) mediated by C3b formation resulting in C5 convertase activity and subsequent assembly of the MAC [12, 13]. IVH presents clinically with decreased hemoglobin levels, increased serum levels of lactate dehydrogenase ([LDH] which is an enzyme released from lysed RBCs) and bilirubin (which is a product of heme catabolism), and an elevated absolute reticulocyte count ([ARC] due to the bone marrow’s compensatory production of RBCs) [3, 14–17]. Due to the chronic hemolysis, individuals with PNH may develop chronic anemia, which in turn can have debilitating consequences including fatigue, dyspnea, and pain, contributing to impaired quality of life (QoL) [18–21]. Patients may also require routine RBC transfusions, depending on their hemoglobin levels [18].

Eculizumab (Alexion Pharmaceuticals, Inc, Boston, MA) is a humanized monoclonal antibody C5 inhibitor and the first complement inhibitor approved for the treatment of PNH [22]. Although eculizumab prevents IVH by binding to C5 and inhibiting the action of C5 convertase, individuals often experience residual RBC lysis (extravascular hemolysis [EVH]) [4]. EVH is mediated by C3b accumulation at the cell surface resulting in opsonization and subsequent erythrocyte breakdown through the action of macrophages [4, 13]. The rate at which EVH occurs in patients with PNH is difficult to assess in untreated individuals, presumably due to the rapid clearance of PNH RBCs through terminal complement activity with IVH [4]. However, deposition of C3 fragments on PNH RBCs is detectable in patients treated with eculizumab, suggesting that EVH is a source of ongoing hemolysis in C5 inhibitor-treated patients with PNH [4, 23]. In fact, despite eculizumab treatment, up to 72% of patients continue to experience residual anemia, and up to 36% of patients require more than 1 RBC transfusion per year [23]. Based on the literature, 5–30% of patients experience breakthrough hemolysis with eculizumab treatment [24–26]. One study suggests that only 13% of patients on eculizumab can be classified as complete responders, who are defined as patients with normal hemoglobin levels (for a duration of > 6 months) and experience a decrease in PNH-related symptoms (thromboses, smooth muscle dystonia), achieve complete transfusion independence, and have LDH levels < 1.5 times the upper limit of normal (ULN) [23, 27]. An updated hematologic response categorization to eculizumab based on hemoglobin levels, residual hemolysis, and transfusion requirements from a multicenter study suggests that ~ 21% of eculizumab patients can be classified as complete responders whereas > 38% of patients only achieve a partial or minor hematologic response [28].

A second humanized monoclonal antibody C5 inhibitor, ravulizumab (Alexion Pharmaceuticals), was approved by the Food and Drug Administration (FDA) in December 2018 for the treatment of PNH. While ravulizumab shares the same structural backbone and mechanism of action as eculizumab, it has a 4-amino acid substitution that extends its half-life [29, 30]. Studies have shown that ravulizumab is non-inferior to eculizumab in transfusion avoidance and hemoglobin stabilization [29]. However, similar to eculizumab, patients may still experience residual RBC lysis [31].

The recently FDA- and the European Medicines Agency (EMA)-approved complement inhibitor pegcetacoplan (USA: Empaveli™; European Union: Aspaveli®; APL-2) is a pegylated 15-amino acid cyclic peptide that binds to C3 and C3b, exerting broad inhibition of the complement cascade, and thus provides improved control of complement-mediated hemolysis [32, 33]. Here we present the results of the PADDOCK and PALOMINO trials, which assessed the safety, tolerability, efficacy, pharmacokinetics (PK), and pharmacodynamics (PD) of pegcetacoplan in complement inhibitor-naïve patients (PNH patients who have not previously received treatment with the C5 inhibitors eculizumab or ravulizumab).

## Methods

### Study design

**Fig. 1:**
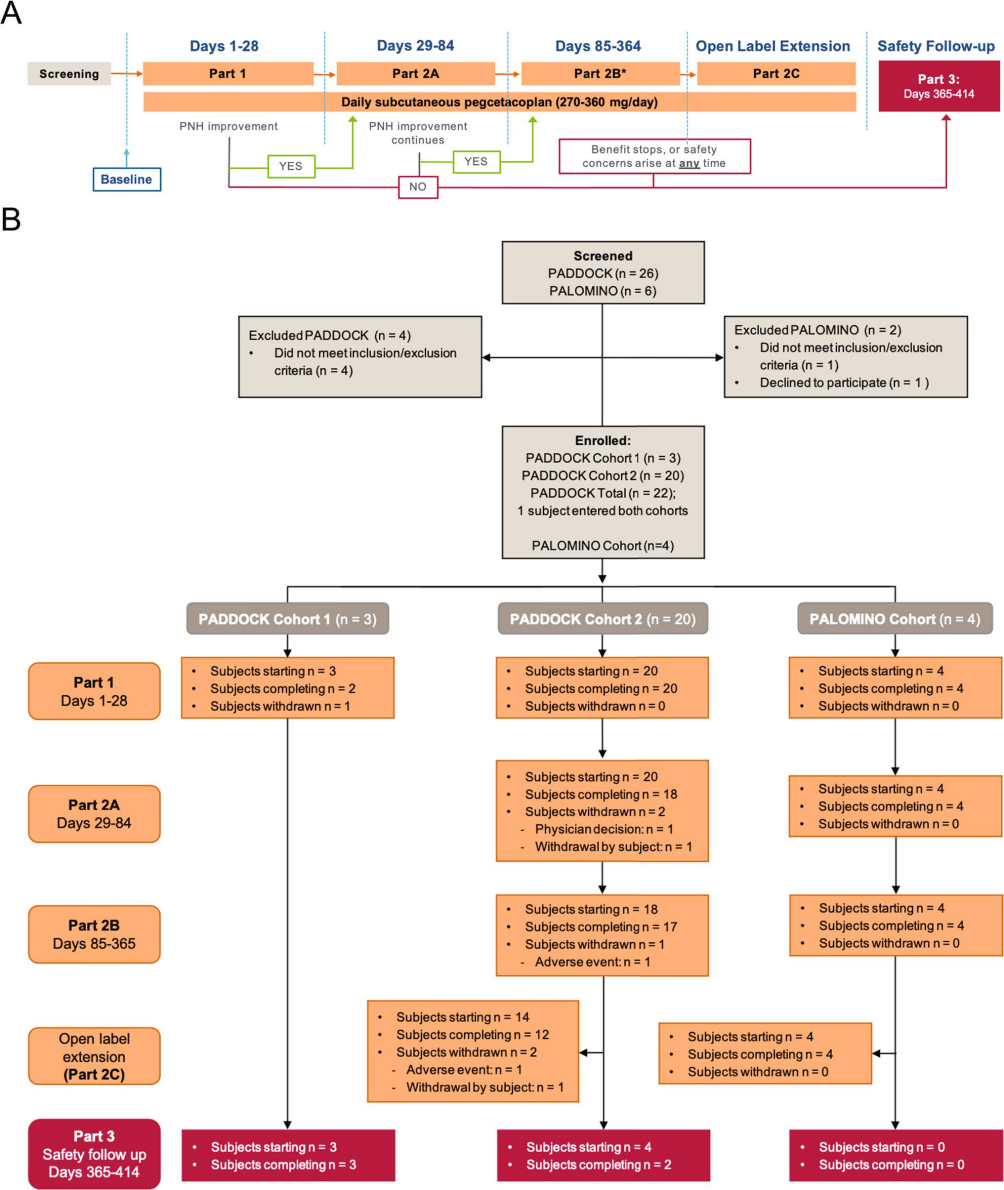
Study design and disposition for PADDOCK and PALOMINO. **A** Study design and endpoints investigated; subjects in the PADDOCK trial could participate in cohort 1 and cohort 2. One subject participated in both cohorts. Cohort 1: daily subcutaneous 180 mg pegcetacoplan (*n* = 3). Cohort 2: daily subcutaneous 270–360 mg pegcetacoplan (*n* = 20) was initiated after the Safety and Monitoring Committee had determined that pegcetacoplan has an acceptable safety and tolerability profile in cohort 1. Clinical benefit was evaluated throughout the trial and if a patient did not experience PNH symptom improvement, the subject would be withdrawn from the study and entered the follow-up period (Part 3). **B** CONSORT diagram for PADDOCK and PALOMINO. *Asterisk*: Subjects who transitioned to either the extension study or Part 2C completed the day 365 visit for Part 3 while continuing to receive pegcetacoplan. For these subjects, the day 365 visit was also the first visit of the open-label extension study or Part 2C and these subjects did not complete Part 3. *Dagger*: All subjects were offered to enroll in an extension study and the majority of subjects chose to continue

All procedures followed for both trials were in accordance with guidance for Good Clinical Practice, the ethical standards of the responsible committee on human experimentation (institutional and national), and with the Helsinki Declaration of 1975, as revised in 2008. The protocol was approved by the relevant Institutional Review Boards/Ethics Committees. PADDOCK was a phase 1b, open-label, multiple-ascending dose pilot trial (NCT02588833) consisting of 2 cohorts and multiple parts (Fig. 1A) conducted at 9 study centers across the world (Hong Kong [study sites: 2], Malaysia [1], New Zealand [2], Thailand [3], USA [1]). Subjects could be enrolled into both cohorts. Patients self-administered pegcetacoplan subcutaneously (180 mg/day cohort 1; 270 mg/day cohort 2). In cohort 2, pegcetacoplan doses could be increased up to 360 mg/day, if indicated by a suboptimal clinical response but acceptable tolerability to treatment. Details on the trial protocol can be found in the Online Resource Methods.

PALOMINO (NCT03593200) was a phase 2a, open-label, multiple-dose single cohort study using the same design and dosing schedule as PADDOCK trial cohort 2 (Fig. 1A) conducted at 3 study centers across 2 countries (Bulgaria [2] and Serbia [1]).

### Patient inclusion/exclusion criteria

PADDOCK and PALOMINO enrolled PNH adult patients aged ≥ 18 years with PNH white blood cell clone sizes of > 10%, who had the following: received a transfusion within 12 months prior to screening; a platelet count of > 30,000/mm^3^; an absolute neutrophil count > 500/mm^3^; and LDH levels ≥ 2 times the ULN at the screening visit. Subjects who had received prior eculizumab treatment were excluded from both trials. Detailed inclusion/exclusion criteria for PADDOCK and PALOMINO are shown in Table 1. Written informed consent was obtained for each trial participant.

**Table 1:** Inclusion and exclusion criteria for PADDOCK (NCT02588833) and PALOMINO (NCT03593200) trials

Inclusion	Exclusion
≥ 18 years old	Prior eculizumab (Soliris) treatment
Diagnosed with PNH (white blood cell clone > 10%)	Hereditary complement deficiency
LDH ≥ 2 times the ULN	History of bone marrow transplantation
Ferritin ≥ LLN and total iron-binding capacity ≤ ULN. If receiving iron supplements, must be stable for 8 weeks prior to enrollment and maintained during the study	Concurrent severe aplastic anemia
Last transfusion within 12 months prior to screening	Participation in any other investigational drug, trial device, or procedure within 30 days
Platelet count of > 30,000/mm^3^	Evidence of QT interval corrected for heart rate using Fridericia’s formula (QTcF), defined as > 450 ms for men and > 470 ms for women
Absolute neutrophil count > 500/µL	Breastfeeding women
Women of childbearing potential had to have a negative pregnancy test and agree to use protocol-defined methods of contraception	History of meningococcal disease
Men had to agree to use protocol-defined methods of contraception and refrain from donating sperm	Active bacterial infection
Current vaccination against *N. meningitidis* types A, C, W, Y, and B; *S. pneumoniae*; and *Haemophilus influenzae* type b (Hib)	Active infection with hepatitis B^a^, hepatitis C^a^, or human immunodeficiency virus^a^
Willing and able to give informed consent	
Weigh > 40 kg^a^ and have a BMI ≤ 38.0 kg/m^2a^	

*BMI*, body mass index; *LLN*, lower limit of normal; *PNH*, paroxysmal nocturnal hemoglobinuria; *ULN*, upper limit of normal

^a^PADDOCK trial inclusion/exclusion criteria only

### Endpoints

For both trials, the primary efficacy endpoints were mean change from baseline (CFB) in hemoglobin, LDH, and haptoglobin levels derived from central laboratories. Primary safety endpoints included the number and severity of treatment-emergent adverse events (TEAEs). Secondary endpoints included the mean CFB in absolute reticulocyte count, total bilirubin level, and Functional Assessment of Chronic Illness Therapy (FACIT)-Fatigue score (for details see Online Resource Methods) [34].

Evaluated PK endpoints included pegcetacoplan serum concentrations over time, and PK assessments were typically taken pre-dose, except on day 1, where the PK sample was taken a minimum of 2.5 h after dosing. For analyses of drug safety and immunogenicity, serum samples were collected throughout each study and tested for anti-drug antibodies by a bioanalytical laboratory.

Exploratory (PD) endpoints assessed were mean CFB in complement parameters (CH50, AP50, and C3), C3 deposition on type II and type III RBCs, clonal distribution of PNH RBCs, PNH granulocytes, and PNH monocytes.

### Statistical analysis

For both trials, absolute values for hemoglobin, LDH, haptoglobin, FACIT-Fatigue score, ARC, total bilirubin, and mean pegcetacoplan serum concentrations were summarized. Overall, mean absolute values ± SE were plotted for PADDOCK cohort 2 and all PALOMINO subjects. Where appropriate, central laboratory upper or lower limits of normal (ULN or LLN) were included as reference values in the figures. All 12-month transfusion history data and on-study transfusion data were provided. Exploratory PD endpoint absolute values were summarized using mean and standard deviation (SD). No formal statistical comparison was made between the PADDOCK and PALOMINO trials.

All enrolled PADDOCK (*n* = 22) and PALOMINO (*n* = 4) subjects were included in the respective safety analyses because all received ≥ 1 dose of pegcetacoplan. The number of subjects with TEAEs and treatment-related TEAEs was presented by System Organ Class and preferred term; no formal statistical analyses of TEAEs were performed.

## Results

### Subject demographics and baseline characteristics

Of the 26 subjects screened in the PADDOCK trial, 22 (9 females, 13 males) met the entry criteria; 3 were enrolled in cohort 1 and 20 in cohort 2 (1 subject entered both cohorts) (Fig. 1B). Overall, 17 subjects completed the trial protocol between December 2015 and August 2019 across 9 study centers. For PALOMINO, 6 subjects were screened, and 4 subjects (3 females, 1 male) were enrolled across 3 study centers (Fig. 1B) between August 2018 and October 2019. All 4 PALOMINO subjects completed the study protocol.

Demographics and baseline characteristics data for both PADDOCK and PALOMINO trials are presented in Table 2. Baseline values were generally similar across the 2 trials.

**Table 2:** Demographics and baseline patient characteristics

**Parameter**	**PADDOCK** (phase 1b)			**PALOMINO** (phase 2a)
	**Cohort 1,** *n* = 3 (180 mg)	**Cohort 2,** *n* = 20 (270 mg)	**Combined overall,** *n* = 22	*n* = 4 (270 mg)
**Age,** years				
Mean age (range)	42.0 (30, 61)	43.2 (22, 67)	42.2 (22, 67)	30.8 (22, 47)
**Sex,** *n* (%)				
Male	2 (66.7)	11 (55.0)	13 (59.1%)	1 (25.0)
Female	1 (33.3)	9 (45.0)	9 (40.9%)	3 (75.0)
**Race,** *n* (%)				
Asian	0	15 (75.0)	15 (68.2)	0
White	3 (100)	1 (5.0)	3 (13.6)	4 (100)
Native Hawaiian or Other Pacific Islander	0	1 (5.0)	1 (4.5)	0
Maori	0	1 (5.0)	1 (4.5)	0
Other	0	2 (10.0)	2 (9.1)	0
**Body mass index,** mean (SD), kg/m^2^	26.50 (4.37)	24.68 (4.48)	25.01 (4.49)	27.20 (5.24)
**Time since PNH diagnosis,** mean (range), year	16.74 (1.1, 38.8)	12.77 (1.3, 38.8)	12.13 (1.1, 38.8)	2.87 (0.8, 7.0)
**Platelets,** mean (SD) × 10^9^/L	ND	159.1 (67.54)	ND	269.0 (85.85)
**Number of RBC transfusions** in the year prior to first enrolled cohort, mean (range)^a^	6.0 (3, 10)	4.8 (0, 13)	5.0 (0, 13)	5.0 (2, 9)
**Pre-dose hemoglobin,** mean (SD), g/dL [NRR: females 12–16, males 13.6–18]	9.00 (1.31)	8.38 (1.83)	8.51 (1.78)	7.73 (0.86)
**Pre-dose haptoglobin,** mean (SD), g/L [NR: 0.14–2.58]	0.04 (0.00)	0.04 (0.01)	0.04 (0.01)	0.10 (0.00)
**Pre-dose LDH**, mean (SD), U/L [NRR: 113–226]	1970.0 (598.36)	2388.8 (1014.13)	2354.9 (987.95)	2548.8 (631.12)
**Pre-dose total bilirubin,** mean (SD), mg/dL [NRR: 1.7–18.8]	30.7 (12.86)	42.6 (25.59)	41.1 (25.01)	30.85 (16.29)
**Pre-dose ARC,** mean (SD), × 10^9^/L [NRR: females, males, 30–100]	196.7 (85.17)	194.9 (62.16)	198.2 (62.95)	238.3 (91.01)
**Pre-dose FACIT-Fatigue score,** mean (SD)	34.7 (4.16)	34.6 (10.56)	35.0 (9.98)	40.5 (4.04)
**C3 complement**, g/L	0.84 (0.11)	0.91 (0.20)	0.92 (0.19)	1.10 (0.14)
**CH50** [total hemolytic complement assay]^b^	609.00 (110.31)	589.00 (91.75)	594.40 (91.37)	58.13 (18.32)
**AP50** [complement alternative pathway assay]^c^	0.84 (0.02)	0.84 (0.27)	0.85 (0.26)	2.958 (0.81)
**PNH granulocytes,** mean (SD), % FLAER	ND	89.25 (12.97)	ND	71.45 (9.01)
**PNH monocytes,** mean (SD), % FLAER	ND	93.33 (6.49)	ND	93.63 (5.00)
**Clonal distribution of type II and type III PNH RBCs,** % mean (SD)	ND	39.81 (21.39)	ND	42.2 (8.06)
**C3 deposition on type II and type III PNH RBCs,** % mean (SD)	ND	1.51 (1.80)	ND	4.3 (6.10)

*ARC*, absolute reticulocyte count; *FACIT-Fatigue*, Functional Assessment of Chronic Illness Therapy Fatigue scale; *FLAER*, fluorescein-labeled proaerolysin; *LDH*, lactate dehydrogenase; *ND*, not determined; *PNH*, paroxysmal nocturnal hemoglobinuria; *RBC*, red blood cell; *SD*, standard deviation

^a^Number of transfusions in the last 12 months prior to randomization

^b^Different assays used for the analysis of CH50 between PADDOCK and PALOMINO studies. For PADDOCK, CH50 reported as an activity unit (U); for PALOMINO, CH50 reported as a concentration unit (U Eq/mL)

^c^AP50 reported without a unit since a normalized value against control was used

### Safety

Overall, 19 subjects (86.4%) from the PADDOCK trial experienced at least 1 TEAE, with a total of 143 TEAEs reported (Table 3). In addition, 7 subjects reported serious adverse events (SAEs, 13 events) during the trial. The majority of TEAEs (129) and SAEs (12) occurred between part 1 and part 2B. No TEAEs were reported during part 3. Ten subjects (45.5%) experienced 35 TEAEs that were at least possibly related to study drug (Online Resource Table 1). Of these, the most common treatment-related TEAEs were injection site erythema (4 subjects [18.2%]; 9 events), rash maculo-papular (2 subjects [9.1%]; 2 events), skin hypopigmentation (1 subject [4.5%]; 6 events), and hypokalemia (2 subjects [9.1%]; 2 events) (Online Resource Table 1). Two subjects in the PADDOCK trial experienced breakthrough hemolysis (5 events total); 1 patient experienced a single breakthrough hemolysis event related to a fatal treatment-emergent SAE of aplastic anemia (details described later; considered unrelated to pegcetacoplan), and the remaining 4 breakthrough hemolysis events occurred in 1 patient following infection (pneumonia) who received a pegcetacoplan dose increase after the third event (additional details are provided in the Online Resource Results: Breakthrough Hemolysis Section). Three subjects discontinued the PADDOCK trial due to adverse events (moderate treatment-emergent SAE of hypersensitivity, severe treatment-emergent SAE of abdominal neoplasm [ovarian adenocarcinoma], and fatal treatment-emergent SAE of aplastic anemia). The subject experiencing the fatal treatment-emergent SAE of aplastic anemia died 56 days after discontinuing pegcetacoplan treatment, and the event was considered unrelated to pegcetacoplan treatment (Table 3).

**Table 3:** Treatment-emergent adverse events for both PADDOCK and PALOMINO trials

Event	PADDOCK (phase 1b)			PALOMINO (phase 2a)
	**Cohort 1,** *n* = 3 (180 mg)	**Cohort 2,** *n* = 20 (270 mg)	**Combined overall,** *n* = 22	*n* = 4 (270 mg)
**Serious AE, *n* (%)**	1 (33.3)	6 (30.0)	7 (31.8)	1 (25.0)
**AEs leading to study drug discontinuation**	1 (33.3)	2 (10.0)	3 (13.6)	0
**AEs leading to death**	0	1 (5.0)^a^	1 (4.5)^a^	0
**Any TEAE, *n* (%)**	2 (66.7)	18 (90.0)	19 (86.4)	3 (75.0)
**Treatment-related TEAEs, *n* (%)**	2 (66.7)	9 (45.0)	10 (45.5)	2 (50.0)

A subject could be counted more than once per category in total events, but when counting total unique AEs, a subject was only counted once for each AE Preferred Term

*AE*, adverse event; *TEAE*, treatment-emergent adverse event

^a^One subject died during the study 56 days after discontinuing pegcetacoplan treatment. The subject had a fatal treatment-emergent SAE of aplastic anemia, which was deemed *not* related to study drug administration

TEAEs were reported in 3 of the 4 PALOMINO trial subjects, with the most common treatment-related TEAEs being injection site reactions and skin erythema (Table 3, Online Resource Table 2). No cases of breakthrough hemolysis were reported in PALOMINO and no TEAEs resulted in death or study drug discontinuation in PALOMINO. There was 1 SAE reported during the study (rib fracture) that was considered not related to study drug.

Before subjects entered the trial, 9.1% of PADDOCK and 75% of PALOMINO patients experienced thrombotic episodes (Online Resource Table 3). However, no thrombotic TEAEs were observed during either trial for the observational period of 365 days (Online Resource Table 3). Only 2 serum samples, each from a distinct patient, out of the 212 samples tested (PADDOCK, *n* = 177 samples; PALOMINO, *n* = 35 samples) had a confirmed positive result for anti-pegcetacoplan peptide antibodies (see details in Online Resource Results: Anti-drug Antibodies Section).

### Primary endpoints and hematologic improvements over time

**Fig. 2:**
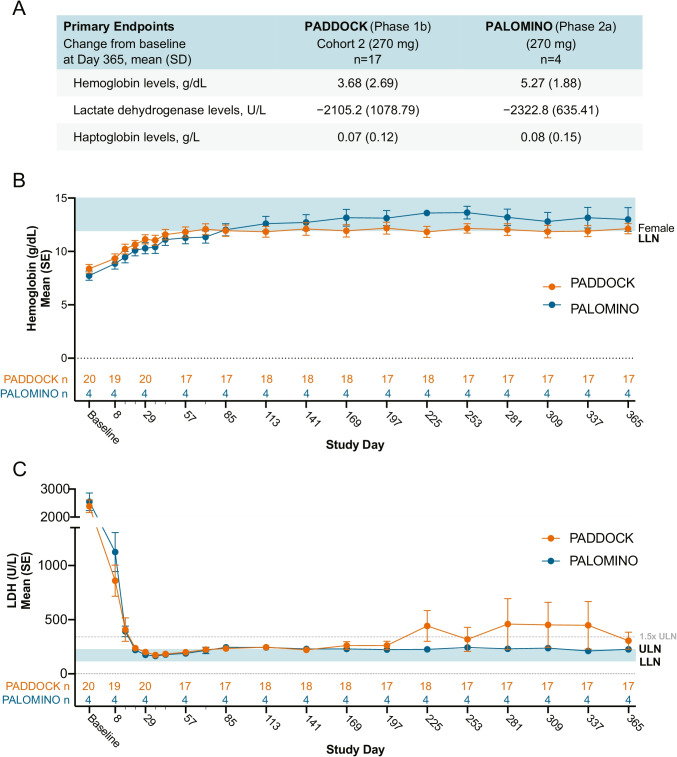
Primary endpoint findings and hematologic improvements over time in subjects with PNH treated with pegcetacoplan. **A** Primary endpoints for the PADDOCK and PALOMINO studies; mean (SD) change from baseline at day 365 in hemoglobin, LDH, and haptoglobin levels.** B** Pegcetacoplan rapidly reduced hemoglobin levels. Mean hemoglobin levels over time are depicted for cohort 2 of the PADDOCK trial and all 4 PALOMINO subjects. The blue shaded area indicates the normal range of hemoglobin levels with the bottom of the shaded area depicting the lower limit of normal (LLN) for female patients [NRR: females 11.9–16]. Smaller dashes on the *x*-axis indicate additional time points investigated (days 15, 22, 36, 43, and 71), which were left off the *x*-axis to not overcrowd the axis. *N*’s for both trials are listed immediately above the *x*-axis. **C** Pegcetacoplan dosing rapidly reduced LDH levels. Mean LDH levels over time are depicted for cohort 2 of the PADDOCK trial and all 4 PALOMINO subjects. The *y*-axis is split to better show differences at lower LDH levels. The blue shaded area depicts the normal range 113–226 U/L for LDH with the ULN, LLN, and 1.5 × ULN (339 U/L) marked. Slight increases in mean LDH levels on day 225 in the PADDOCK trial were due to 3 patients that experienced an increase in LDH levels on this day. One of these patients continued to exhibit elevated LDH levels on day 309 and day 337. These LDH increases most likely result from the patients experiencing adverse events, or serious adverse events such as the abdominal neoplasm and hemolysis during these study days. LDH, lactate dehydrogenase; LLN, lower limit of normal; NRR, normal reference range; SD, standard deviation; SE, standard error, ULN, upper limit of normal

In the PADDOCK and PALOMINO trials, daily subcutaneous administration of pegcetacoplan contributed to hematologic improvements. Specifically, primary endpoint results demonstrated that hemoglobin levels increased from baseline (mean change from baseline at day 365: PADDOCK, 3.7 g/dL; PALOMINO, 5.3 g/dL) and LDH levels decreased from baseline (mean change from baseline at day 365: PADDOCK, − 2105.2 U/L; PALOMINO, − 2322.8 U/L) (Fig. 2A). Patients from both studies demonstrated minor improvements in the primary endpoint of the mean change from baseline in haptoglobin levels with mean increases from baseline of 0.07 g/L (PADDOCK) and 0.08 g/L (PALOMINO) at day 365 (Fig. 2A).

Analyses of mean hematologic values over time indicated that daily subcutaneous administration of pegcetacoplan resulted in rapid improvements in mean hemoglobin and LDH levels that were sustained throughout the PADDOCK and PALOMINO studies. Pegcetacoplan contributed to an increase in mean hemoglobin levels from below the LLN (normal range: 11.90–18.00 g/dL) at baseline (mean 8.38 g/dL) to 10.64 g/dL on day 22, and within the normal range by day 85 (11.95 g/dL) (Fig. 2B). This increase was maintained for the duration of the trial (day 365: 12.14 g/dL). Similar mean hemoglobin level increases were observed in PALOMINO subjects (day 22 mean: 10.10 g/dL; day 85: mean 12.03 g/dL; day 365: mean 13.00 g/dL) (Fig. 2). Eight subjects (47.1%) in the PADDOCK trial and 3 subjects (75.0%) in the PALOMINO trial had hemoglobin values within the normal range at day 365. Patients treated with pegcetacoplan display rapid and sustained improvements in mean LDH levels, which at baseline were ≥ 8 times the ULN (normal range: 120.0–250.0 U/L; PADDOCK mean: 2354.9 U/L; PALOMINO mean: 2548.8 U/L) (Fig. 2C). For 16 PADDOCK subjects (80.0%), mean LDH levels decreased to within normal range by day 22 (mean 237.5 U/L). In PALOMINO, all 4 subjects (mean 226.0 U/L) and, in PADDOCK, 14/17 subjects (mean 306.5 U/L) had LDH levels ≤ 1.5 × the ULN at day 365 (Fig. 2C). Patients in the PADDOCK study demonstrated mean haptoglobin levels at baseline (0.04 g/dL) that were below the LLN (normal range: 0.14–2.58 g/L) and displayed increased mean haptoglobin levels within the normal range by day 22 (0.51 g/L; 65% [*n* = 13] of patients were within the normal range). However, a marginal decrease in mean haptoglobin levels was observed between day 22 and day 365, and mean haptoglobin levels were slightly below the LLN at day 365 (mean 0.11 g/L) of the PADDOCK study (Online Resource Table 4). For PALOMINO subjects, increases in mean haptoglobin levels were above the LLN on day 365 (mean 0.18 g/dL) (Online Resource Table 4).

### Other biochemical indicators of hemolysis

**Fig. 3:**
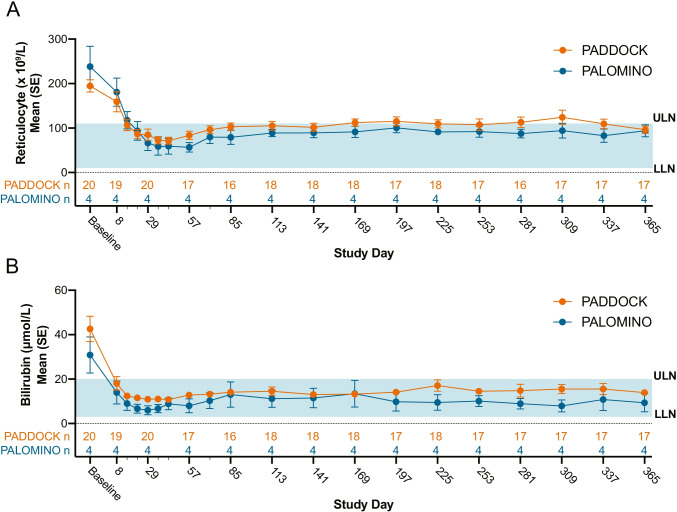
Other biochemical indicators of hemolysis over time in subjects with PNH treated with pegcetacoplan. **A** ARC rapidly decreased upon pegcetacoplan dosing. Mean ARC are depicted for cohort 2 of the PADDOCK trial and all 4 PALOMINO subjects. Blue shaded area depicts the normal range: 10–110 × 10.^9^ cells/L with the ULN and LLN marked. **B** Pegcetacoplan dosing rapidly decreased the total bilirubin levels in both PADDOCK (cohort 2) and PALOMINO (all 4 subjects) trials. Blue shaded area indicates the normal range: 3–20 μmol/L with ULN and LLN marked. Smaller dashes on the *x*-axis indicate additional time points investigated (days 15, 22, 36, 43, and 71), which were left off the *x*-axis to not overcrowd the axis. *N*’s for both trials are listed immediately above the *x*-axis. ARC, absolute reticulocyte count; LLN, lower limit of normal; SE, standard error; ULN, upper limit of normal

Mean ARC levels for PADDOCK and PALOMINO subjects were > 1.5 times the ULN (normal range: 10–110 × 10^9^ cells/L; PADDOCK mean: 194.9 × 10^9^ cells/L; PALOMINO mean 238.3 × 10^9^ cells/L) (Fig. 3A). Upon pegcetacoplan dosing initiation, ARC levels decreased rapidly, resulting in levels that were within normal range on day 365 (PADDOCK mean: 96.4 × 10^9^ cells/L; PALOMINO mean 94.0 × 10^9^ cells/L) (Fig. 3A). At baseline, mean bilirubin was > 2 times the ULN (normal range: 3–20 μmol/L; PADDOCK mean: 41.1 μmol/L; PALOMINO mean: 30.9 μmol/L) (Fig. 3B). Mean bilirubin levels for both PADDOCK and PALOMINO subjects rapidly decreased and were within the normal range by day 8 (PADDOCK mean: 18.0 μmol/L; PALOMINO mean: 14.0 μmol/L) (Fig. 3B). Overall, the decrease in hemolytic markers, LDH and bilirubin, and increased hemoglobin levels suggest that pegcetacoplan protects PNH cells from hemolysis.

### Transfusion requirements

The number of RBC transfusions required before pegcetacoplan treatment was greater than after treatment initiation. In cohort 2 of the PADDOCK trial, 18/20 (90.0%) subjects received ≥ 1 transfusion in the 12 months prior to screening, with 11 subjects receiving > 4 transfusions over this period. Following the initiation of pegcetacoplan dosing, 7/20 PADDOCK subjects (35.0%) required transfusions. It is important to note that for 2 of these subjects, the only transfusions required were given on days 3 and 15, which was prior to pegcetacoplan reaching steady-state serum concentration. Transfusions administered once pegcetacoplan had reached a steady-state concentration in the remaining 5 subjects were associated with SAEs. Overall, 13/20 (65.0%) PADDOCK subjects were transfusion-free following 365 days of pegcetacoplan dosing (Online Resource Table 4). All 4 PALOMINO subjects were transfusion-dependent prior to pegcetacoplan dosing, with a transfusion history ranging from 2 to 9 RBC transfusions in the 12 months prior to entering the trial. All 4 subjects were transfusion-free at day 365 following pegcetacoplan dosing (Online Resource Table 4).

### PK analyses and exploratory endpoints

PK analyses revealed that for the 17 PADDOCK subjects and the 4 PALOMINO subjects who received ≥ 270 mg/day for ≥ 364 days in the trial, serum pegcetacoplan concentrations appeared to reach steady state between 29 and 43 days from the first dose (Online Resource Fig. 1). These results demonstrate further that sustainable therapeutic concentrations of pegcetacoplan were maintained through the treatment periods of the 2 trials.

Pegcetacoplan treatment resulted in an increase in mean C3 levels in the PADDOCK trial (Online Resource Table 4). Mean C3 levels increased to more than 200% of the baseline value starting at day 43 through the rest of the respective treatment period, indicating robust and sustained target C3 engagement and inhibition. Conversely, mean AP50 levels decreased from baseline during the PADDOCK trial period, with a maximum decrease of 70% from baseline on day 29. Subsequently, AP50 levels increased slightly to half of the baseline value at day 160 and remained at a similar level until day 365 (Online Resource Table 4). No significant changes in CH50 and proportions of PNH granulocytes and monocytes were observed throughout the trial (Online Resource Table 4). Similar results were seen for these exploratory markers in the PALOMINO trial (Online Resource Table 4).

The sum of mean clonal distributions of type II and type III PNH RBCs doubled between baseline and day 365 (baseline: PADDOCK mean: 39.8%; PALOMINO mean: 42.2% versus day 365: PADDOCK mean: 84.0%; PALOMINO mean: 93.0%) (Online Resource Table 4). This suggests that pegcetacoplan was effective in protecting PNH type II and type III RBCs from complement-mediated lysis. Additionally, C3 deposition on type II and type III RBCs (an indicator of opsonization predisposing to EVH) decreased after pegcetacoplan treatment in both the PADDOCK and PALOMINO trials (Online Resource Table 4).

### Quality of life

**Fig. 4:**
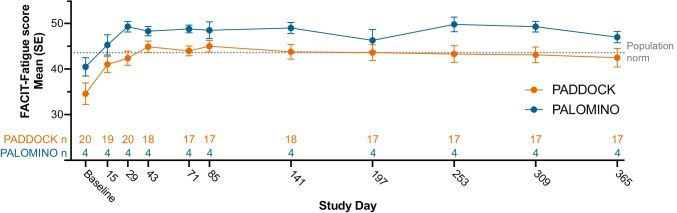
Mean Functional Assessment of Chronic Illness Therapy (FACIT)-Fatigue scores over time in subjects with PNH treated with pegcetacoplan. Mean FACIT-Fatigue scores are depicted for cohort 2 of the PADDOCK trial and all 4 PALOMINO subjects. Both PADDOCK and PALOMINO mean scores were below the population norm (43.6; gray dotted line) [35] at baseline, but rapidly increased to within or above the population norm. Both trials demonstrate a greater than 3-point increase in the FACIT-Fatigue score, which is considered clinically meaningful [35]. *N*’s for both trials are listed immediately above the *x*-axis. FACIT, Functional Assessment of Chronic Illness Therapy; SE, standard error

The FACIT-Fatigue score was used to assess the perceived fatigue levels reported by subjects during the trial period. At baseline, both PADDOCK and PALOMINO subjects, on average, scored below the population norm on the FACIT-Fatigue scale (population norm 43.6 [35]; PADDOCK mean: 35.0; PALOMINO mean: 40.5). Upon pegcetacoplan dosing initiation, FACIT-Fatigue scale scores increased, where PADDOCK subjects scored on average similar to the population norm on day 43 (mean: 44.9) and maintained this increase through day 365 (mean 48.5) (Fig. 4). Mean scores increased by greater than 3 points which is deemed clinically relevant [35]. PALOMINO subjects averaged around the population norm as early as day 15 (mean 45.3), which was maintained throughout the trial (47.0).

## Discussion

**Fig. 5:**
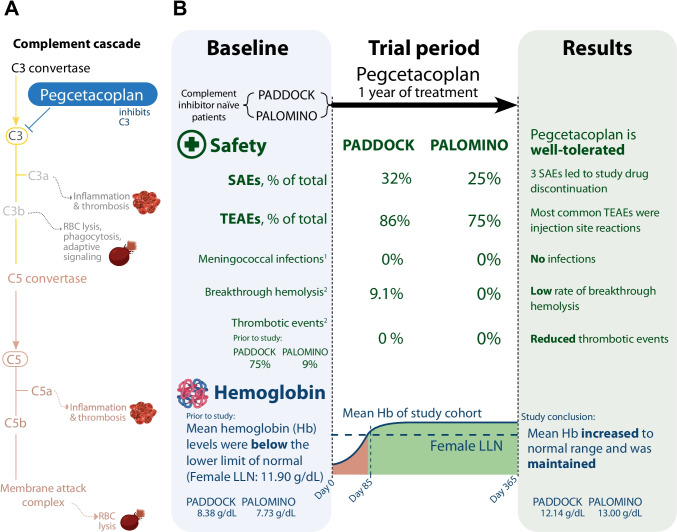
Pegcetacoplan improves hemolysis markers in patients with PNH. **A** The complement cascade and location of pegcetacoplan inhibition at the level of C3 and its downstream effects. **B** Summarized results from baseline to the 1-year endpoint for specific safety events as well as hemoglobin levels. The thrombosis, red blood cell (RBC) lysis, and hemoglobin icons were created using biorender.com. Footnotes: ^1^Adverse event of special interest; ^2^treatment-emergent adverse event. SAE, serious adverse event; TEAE, treatment-emergent adverse event

The results of the PADDOCK and PALOMINO trials demonstrate that pegcetacoplan treatment improved hematologic parameters by controlling hemolysis in patients with PNH. Pegcetacoplan differs from other approved PNH therapies as it inhibits the complement system at the level of C3, rather than C5, which acts downstream from C3 (Fig. 5A) [32, 33]. Pegcetacoplan treatment in complement inhibitor-naïve subjects with PNH resulted in rapid and durable changes to normal levels of hemoglobin, LDH, ARC, and total bilirubin. Hemoglobin levels rapidly increased from baseline to within the normal range; this increase was maintained for the duration of the trials and was consistent with the decrease in hemolytic markers. Additionally, pegcetacoplan treatment rendered the majority of patients that were previously transfusion-dependent, transfusion-free, and caused clinically relevant improvements from baseline in the FACIT-Fatigue score (> 3-point changes in the FACIT-Fatigue score are considered clinically meaningful [35]), thereby suggesting that pegcetacoplan treatment improved PNH patients’ QoL. Overall, pegcetacoplan was effective, well tolerated, and had an acceptable safety profile.

Broad inhibition of the complement system leads to a theoretical increased risk of encapsulated bacterial infections [36, 37]. In particular, the inhibition of C5 can lead to an increased risk of *Neisseria* sp. infections [36, 37]. However, no cases of meningococcal infection were observed for the duration of the PADDOCK/PALOMINO trials (365 days). One subject (5.0%) of the PADDOCK trial experienced a treatment-related TEAE classified as System Organ Class “infections and infestations,” which was associated with an upper respiratory tract infection. No subjects from the PALOMINO trial had a treatment-related TEAE classified within this class “infections and infestations.” An increased risk of encapsulated bacterial infections was not identified in either trial, although participants were required to be vaccinated against *N. meningitidis* types A, C, W, Y, and B; *Streptococcus pneumoniae*; and *Haemophilus influenzae* type b (Hib) at the beginning of the trial, and also received prophylactic antibiotic therapy throughout the study. As expected, due to the introduction of frequent self-administered subcutaneous injections, injection site reactions were the most frequently reported adverse events in the PADDOCK/PALOMINO trials.

Thrombotic episodes are a significant problem for PNH patients, and up to 10% will present with thrombosis, although the incidence increases over the disease course [38]. While treatment with eculizumab has reduced the incidence of thrombosis, it has not abolished the occurrence of these events [25, 39, 40]. Here we report that no thrombotic episodes were observed throughout the 365-day study protocol of either the PADDOCK or PALOMINO trial, although patients in both trials reported having experienced thrombotic events in their medical history (PADDOCK: 9.0%; PALOMINO 75.0%; Fig. 5B). However, long-period observations are required in order to confirm long-term reduction in thrombotic episodes in pegcetacoplan-treated patients with PNH. Overall, data from both PADDOCK and PALOMINO trials demonstrate that subcutaneous pegcetacoplan dosing over the study period (365 days) is well tolerated and safe, as relatively few severe TEAEs were reported.

Until May 2021, the standard of care for patients with PNH were 2 anti-C5 antibodies (eculizumab and ravulizumab), which inhibit the formation of the MAC. The hematologic response to eculizumab treatment was evaluated using a response criteria involving improvement in anemia, PNH symptoms, and thrombosis [27]. DeZern et al. found that only roughly 13% of patients treated with eculizumab could be classified as complete responders (defined as normal hemoglobin levels for age and sex for more than 6 months, decrease in PNH-related symptoms, including thromboses and smooth muscle dystonia; complete transfusion independence; and LDH < 1.5 times the upper limit of normal) [27]. Debureaux et al. estimate that approximately 21% of eculizumab-treated patients are complete responders based on a simplified classification (hemoglobin levels, residual hemolysis, and transfusion requirement) [28]. Approximately 53% of patients on eculizumab therapy demonstrated partial results, such as reduced LDH levels and decreased transfusion needs, and therefore could be classified as good partial responders [27]. However, 33% of patients treated with eculizumab experienced persistent symptoms and had unchanged transfusion needs and, thus, were suboptimal responders to eculizumab therapy [27]. Uncontrolled EVH can be significant in a subset of eculizumab-treated patients with PNH and is considered to be the principal contributor to the lack of complete eculizumab response in most patients [4]. Furthermore, in clinical studies comparing eculizumab to ravulizumab, ravulizumab was demonstrated non-inferior to eculizumab, and as it also targets the C5 complement cascade step, it is similarly unable to prevent EVH. Together, these data suggest that neither C5 inhibitor can fully control the disease.

The trials described herein show that treatment of complement inhibitor-naïve patients with the recently FDA-approved C3 inhibitor pegcetacoplan results in hemoglobin levels that are within the normal range by day 85. Hemoglobin levels were sustained throughout both studies, demonstrating that pegcetacoplan is able to control hemolysis events in most patients. This is supported by the fact that no cases of breakthrough hemolysis were observed in the PALOMINO trial. Two subjects in PADDOCK experienced breakthrough hemolysis; however, none of these events led to study drug discontinuation. Additionally, LDH levels (a marker of IVH) were within the normal range by day 22 in both trials and were sustained for most patients throughout the trials. Slight increases in mean LDH levels on day 225 in the PADDOCK trial were due to 3 patients who experienced an increase in LDH levels on this day (1 of these patients exhibited elevated LDH levels also on days 309 and 337). Overall, pegcetacoplan was effective in controlling IVH. Of note, PADDOCK patients displayed variable haptoglobin levels during the study and mean haptoglobin levels that were slightly below the LLN at day 365. It is difficult to speculate why mean haptoglobin levels were variable in these patients while other biological markers of hemolysis improved; however, haptoglobin levels can vary with infection, inflammation, genetic polymorphisms, and, in some cases, blood transfusions [41–44]. Therefore, changes in haptoglobin levels may not serve as an informative indicator for some patients with PNH.

We additionally demonstrate that C3 deposition on type II and type III RBCs, an indicator of opsonization predisposing to EVH, was decreased following pegcetacoplan treatment. This suggests that pegcetacoplan protects type II and type III PNH RBCs from complement-mediated attack and hemolysis. The decrease in complement parameter AP50 and increase in C3 are indicative that repeated dosing of pegcetacoplan leads to a persistent, yet partial, inhibition of alternative complement pathway hemolytic activity while still achieving control of PNH RBC lysis. CH50 levels did not exhibit meaningful changes and proportions of PNH granulocytes and monocytes were minimally changed during the course of the trials. Overall, these data demonstrate that pegcetacoplan is a viable treatment option for patients with PNH since these and a previous trial (PHAROAH, NCT02264639) demonstrated that pegcetacoplan is able to control hemolysis events and improve hematologic parameters [33]. Further, data from the PEGASUS trial (NCT03500549) at 16 weeks demonstrate that pegcetacoplan was superior to eculizumab in improving hemoglobin levels and other clinical and hematologic parameters in patients with PNH [45].

The improvements in hemoglobin levels and the decreases in hemolysis markers correlate well with observed improvements in patient-reported symptoms. These trials reveal that pegcetacoplan also has the potential to increase QoL and fatigue symptoms as clinically meaningful increases (≥ 3 points) in the FACIT-Fatigue score [35] were seen as early as day 15 and were sustained throughout both the PADDOCK and PALOMINO trials. A FACIT-Fatigue score of 43.6 is considered normal as this is the mean reported for healthy adults [34]. At baseline, the mean FACIT-Fatigue score for both PADDOCK and PALOMINO trials was lower than the population norm; however, pegcetacoplan-treated patients achieved similar scores to the population norm as soon as day 15 for PALOMINO and day 43 for PADDOCK. Taken together, these data demonstrate that pegcetacoplan significantly improves the perceived QoL in patients with PNH. However, it is possible that patient-reported fatigue may be an under- or over-estimation because the assessment relies on the patient to self-report their perceived level of fatigue.

Pegcetacoplan offers patients increased flexibility as it can be self-administered at home, in comparison to eculizumab and ravulizumab, which are both administered intravenously [29, 30, 46] and thus require a trained nurse to administer the drug. To reduce the burden of daily pegcetacoplan dosing on subjects (as done in the phase 1 and 2 PHAROAH [33], PADDOCK, and PALOMINO trials) and to promote dosing compliance, the effectiveness of less frequent dosing regimens, such as twice-weekly dosing, was investigated. A twice weekly dose of 1080 mg was selected as the dosing regimen for the phase 3 trials (PEGASUS [45] and PRINCE) on the basis of preliminary PK modeling, which predicted a pegcetacoplan serum level for this regimen between those observed for the 270 and 360 mg daily dose regimens, potentially striking a balance between identifying a minimally effective dose and optimizing the potential clinical response for a broad PNH patient population.

In conclusion, the PADDOCK and PALOMINO trials demonstrate that daily subcutaneous pegcetacoplan injection is an effective treatment for PNH-related anemia in patients not previously treated with C5 inhibitor therapy. Overall, pegcetacoplan demonstrated long-term (365-day) safety, significantly improved and brought hemoglobin and LDH levels (primary efficacy endpoints) to within the normal range, and improved the QoL in these trials. Here, we show that the newly FDA- and EMA-approved C3 inhibitor pegcetacoplan is able to control hemolysis in the majority of complement inhibitor-naïve patients and improve hematologic parameters, thus demonstrating that it is a viable treatment option for treatment-naïve patients with PNH. The PADDOCK/PALOMINO trials support further evaluation of pegcetacoplan in the recently completed phase 3 PRINCE trial in complement inhibitor-naïve patients.

## Supplementary Information

Below is the link to the electronic supplementary material.

Supplementary file1 (DOCX 175 KB)

## Data Availability

All relevant summary data are provided in the manuscript text, tables, and figures.
