# Supplementary material for: Inhibition of C3 with pegcetacoplan results in normalization of hemolysis markers in paroxysmal nocturnal hemoglobinuria
Source: Ann Hematol. 2022 Jul 22;101(9):1971–86. doi: 10.1007/s00277-022-04903-x (PMC9375762; doi:10.1007/s00277-022-04903-x)
Supplement: Supplementary file 1 — Supplementary file1 (DOCX 175 KB) [file 277_2022_4903_MOESM1_ESM.docx]

**Title:** Inhibition of C3 with Pegcetacoplan Results in Normalization of Hemolysis Markers in Paroxysmal Nocturnal Hemoglobinuria

**Journal:** Annals of Hematology

**Authors:** Raymond S.M. Wong, MD, MRCP, FRCP, Humphrey W.H. Pullon, FRACP, FRCPA, Ismail Amine, MD, Andrija Bogdanovic, MD, PhD, MSc, Pascal Deschatelets, PhD, Cedric G. Francois, MD, PhD, Kalina Ignatova, MD, Surapol Issaragrisil, MD, Pimjai Niparuck, MD, Tontanai Numbenjapon, MD, Eloy Roman, MD, Jameela Sathar, MD, MRCP, FRCPath, Raymond Xu, PhD, Mohammed Al-Adhami, PhD, Lisa Tan, RN, Eric Tse, MBBS, PhD, FRCP, FRCPath, Federico V Grossi, MD, PhD

**Corresponding author:**

Federico V Grossi, MD, PhD

Address: 100 5th Avenue; Waltham, MA 02451, USA

Email: federico@apellis.com

Phone number: 617-977-5701 extension 8101

**ONLINE RESOURCE METHODS AND DATA**

[Online Resource Methods: Pegcetacoplan concentration and detailed trial protocol 4](#_Toc101438866)

[Pegcetacoplan administration and dosing 4](#_Toc101438867)

[Trial Protocol 4](#_Toc101438868)

[Functional Assessment of Chronic Illness Therapy (FACIT)-Fatigue Scale 4](#_Toc101438869)

[Statistical analysis 5](#_Toc101438870)

[Online Resource Results 6](#_Toc101438871)

[Online Resource Table 1: Treatment-related treatment-emergent adverse events by System Organ Class and preferred term for PADDOCK 6](#_Toc101438872)

[Breakthrough Hemolysis 7](#_Toc101438873)

[Online Resource Table 2: Treatment-related treatment-emergent adverse events by system organ class and preferred term for PALOMINO (safety set) 8](#_Toc101438874)

[Anti-drug Antibodies (ADA) 9](#_Toc101438875)

[Online Resource Table 3: Thrombosis history for PADDOCK and PALOMINO subjects 10](#_Toc101438876)

[Online Resource Table 4: Additional endpoints for PADDOCK and PALOMINO 11](#_Toc101438877)

[Online Resource Figure 1: Mean serum levels of pegcetacoplan during the PADDOCK and PALOMINO trials 12](#_Toc101438878)

**Investigator Listing PADDOCK**

| **Site** | **Investigator** | **Role** | **# Subjects Enrolled** |
| --- | --- | --- | --- |
| Prince of Wales Hospital,  30-32 Ngan Shing Street, Sha Tin, Hong Kong | Raymond Siu Ming Wong | Principal Investigator | 1 |
| Siriraj hospital  2 Wang Lang Road, Bakkoknoi, 10700 Thailand | Surapol Issaragrisil, MD | Principal Investigator | 2 |
| Ramathibodi Hospital  270 Rama VI road, Ratchatewi, 10400, Thailand | Pimjai Niparuck, MD | Principal Investigator | 3 |
| Phramongkutklao Hospital  315 Ratchatewi road, Ratchatewi, 10400, Thailand | Tontanai Numbenjapon, MD | Principal Investigator | 3 |
| Waikato Hospital DHB  11 Ohaupo Road, Hamilton, 3200, New Zealand | Humphrey Pullon, MBChB | Principal Investigator | 5 |
| Lakes Research, LLC,  5801 NW 151 Street, Suite 302, Miami Lakes, 33014, USA | Eloy Roman, MD | Principal Investigator | 1 |
| Hospital Ampang  Jalan Mewah Utara, Pandan Mewah, Ampang, 68000, Malaysia | Jameela Sathar, MD | Principal Investigator | 4 |
| Canterbury Health Laboratories  524 Hagley Avenue, Christchurch, 8011, New Zealand | Ruth Spearing, MBChB | Principal Investigator | 1 |
| Queen Mary Hospital  102 Pokfulam Road, Hong Kong, Hong Kong | Eric Wai Choi Tse, MBBS | Principal Investigator | 2 |

Total subjects: 22

**Investigator Listing PALOMINO**

| **Site** | **Investigator** | **Role** | **# Subjects Enrolled** |
| --- | --- | --- | --- |
| Clinic of Clinical Hematology  6 Plovdivsko Pole Str, Sofia, 1756, Bulgaria | Kalina Krasteva Ignatova, MD | Principal Investigator | 1 |
| Acibadem City Clinic MHAT Tokuda EAD Sofia Clinic of Hematology  51B Nikola Vaptsarov Blvd, Sofia, 1407 Bulgaria | Ismail Mohamad Amine, MD | Principal Investigator | 1 |
| Clinical Center of Serbia  Dr Koste Todorovića 2 Belgrade, 11000, Republic of Serbia | Andrija Bogdanović, MD, PhD | Principal Investigator | 2 |

Total subjects: 4

Online Resource Methods: Pegcetacoplan concentration and detailed trial protocol

*Pegcetacoplan administration and dosing*

Pegcetacoplan was administered on a scheduled basis either through subcutaneous (SC) injection or SC infusion to achieve intended dose. The concentration of pegcetacoplan in the solution ranged from 40 mg/mL to 150 mg/mL for dose administration. SC injection was used when the dosing volume was less than 3mL, otherwise, SC infusion was used for dose administration.

*Trial Protocol*

Following Part 1, safety data as well as efficacy data were evaluated. Patients in cohort 1 directly proceeded to Part 3 of the study (safety follow-up) and any subject that withdrew from the study (at any point during the protocol) also entered the follow-up period (Part 3). Pegcetacoplan administration was discontinued in Part 3 and subjects were followed for safety for 50 days. Clinical benefit was evaluated throughout the trial and if a patient did not experience PNH symptom improvement, the patient would be withdrawn and enter the follow-up period. During Part 2, cohort 2 patients continued to self-administer pegcetacoplan on a daily basis. After completing Part 2B, subjects could continue treatment with pegcetacoplan by transitioning to an open-label non-randomized, multi-center extension study (NCT03531255) or enter Part 2C if enrollment to the extension study was not yet available. Subjects who elected to enroll in the open-label extension study did not complete Part 3. For subjects entering Part 3 (Days 365-414), pegcetacoplan administration was discontinued and subjects were followed for safety for 50 days.

*Functional Assessment of Chronic Illness Therapy (FACIT)-Fatigue Scale*

The FACIT-Fatigue scale is a 13-item Likert-scale instrument that was self-administered by subjects during clinic visits. A higher FACIT-Fatigue score is correlated with less perceived fatigue by the subject. Additional parameters, including the number of PRBC transfusions per month, were recorded.

*Statistical analysis*

For PADDOCK, the sample size was not based on formal statistical testing and no formal statistical analysis was performed on cohort 1 data. Comparisons to baseline and Figures were created for cohort 2 only as cohort 1 received a different dosing regimen. No imputation of missing data for early termination was performed. Where appropriate, screening values were used as baseline in the event of missing Day 1 measurements as applicable. Missing dates/times for the start/stop of medications and AEs were reviewed on a case-by-case basis for potential imputations.

The goal for PALOMINO was to enroll up to 20 subjects and this sample size was not based on formal statistical testing. Due to enrollment rates and emerging data from other ongoing studies (PRINCE phase 3 NCT04085601), a decision was made to stop enrollment at 4 subjects.

On-site data monitoring for both trials was performed by the sponsor’s designee for the duration of the study. The monitor verified the accuracy and completeness of the eCRF entries, source documents, and other study-related records. The monitor verified the accuracy and completeness of the eCRF entries, source documents, and other study-related records. All relevant summary data is included within the tables in the manuscript and supplement.

Online Resource Results

Online Resource Table 1: Treatment-related treatment-emergent adverse events by System Organ Class and preferred term for PADDOCK

| **PADDOCK**  (Phase 1b) | | | |
| --- | --- | --- | --- |
| **System Organ Class -**  Preferred term | **Cohort 1**  (N=3)  n (%) M | **Cohort 2**  (N=20)  n (%) M | **Total**  (N=22)  n (%) M |
| **Participants with ≥ 1 treatment-related TEAE** | 2 (66.7) 5 | 9 (45.0) 30 | 10 (45.5) 35 |
| **General disorders and administration site conditions** | 1 (33.3) 4 | 6 (30.0) 8 | 6 (27.3) 12 |
| Injection site erythema | 1 (33.3) 4 | 4 (20.0) 5 | 4 (18.2) 9 |
| Injection site induration | 0 | 1 (5.0) 1 | 1 (4.5) 1 |
| Injection site pain | 0 | 1 (5.0) 1 | 1 (4.5) 1 |
| Injection site swelling | 0 | 1 (5.0) 1 | 1 (4.5) 1 |
| **Skin and subcutaneous tissue disorder** | 0 | 5 (25.0) 10 | 5 (22.7) 10 |
| Rash maculo-papular | 0 | 2 (10.0) 2 | 2 (9.1) 2 |
| Erythema | 0 | 1 (5.0) 1 | 1 (4.5) 1 |
| Rash | 0 | 1 (5.0) 1 | 1 (4.5) 1 |
| Skin hypopigmentation | 0 | 1 (5.0) 6 | 1 (4.5) 6 |
| **Investigations** | 0 | 2 (10.0) 3 | 2 (9.1) 3 |
| Alanine aminotransferase increased | 0 | 1 (5.0) 1 | 1 (4.5) 1 |
| Blood alkaline phosphatase increased | 0 | 1 (5.0) 1 | 1 (4.5) 1 |
| Blood lactate dehydrogenase increased | 0 | 1 (5.0) 1 | 1 (4.5) 1 |
| **Metabolism and nutrition disorders** | 0 | 2 (10.0) 2 | 2 (9.1) 2 |
| Hypokalemia | 0 | 2 (10.0) 2 | 2 (9.1) 2 |
| **Respiratory, thoracic and mediastinal disorders** | 0 | 2 (10.0) 2 | 2 (9.1) 2 |
| Productive cough | 0 | 1 (5.0) 1 | 1 (4.5) 1 |
| Rhinorrhea | 0 | 1 (5.0) 1 | 1 (4.5) 1 |
| **Blood and lymphatic system disorders** | 0 | 1 (5.0) 1 | 1 (4.5) 1 |
| Thrombocytopenia | 0 | 1 (5.0) 1 | 1 (4.5) 1 |
| **Eye disorders** | 0 | 1 (5.0) 1 | 1 (4.5) 1 |
| Conjunctival hemorrhage | 0 | 1 (5.0) 1 | 1 (4.5) 1 |
| **Immune system disorders** | 1 (33.3) 1 | 0 | 1 (4.5) 1 |
| Hypersensitivity | 1 (33.3) 1 | 0 | 1 (4.5) 1 |
| **Infections and infestations** | 0 | 1 (5.0) 1 | 1 (4.5) 1 |
| Upper respiratory tract infection | 0 | 1 (5.0) 1 | 1 (4.5) 1 |
| **Musculoskeletal and connective tissue disorders** | 0 | 1 (5.0) 1 | 1 (4.5) 1 |
| Arthralgia | 0 | 1 (5.0) 1 | 1 (4.5) 1 |
| **Renal and urinary disorders** | 0 | 1 (5.0) 1 | 1 (4.5) 1 |
| Hematuria | 0 | 1 (5.0) 1 | 1 (4.5) 1 |

*Abbreviations: TEAE: treatment-emergent adverse event; M: number of events; N: overall cohort size; n: number of patients*

Breakthrough Hemolysis

Two subjects in the PADDOCK study experienced breakthrough hemolysis (5 events total). One patient in cohort 2 experienced a single breakthrough hemolysis event related to a severe adverse event of aplastic anemia. This patient died 56 days after discontinuing pegcetacoplan treatment, and the occurrence of aplastic anemia was considered unrelated to pegcetacoplan treatment. The remaining 4 breakthrough hemolysis events occurred in a single cohort 2 patient; event 1 occurred when the patient contracted pneumonia, and the remaining three breakthrough hemolysis events occurred as follows: event 2 occurred two weeks after the resolution of pneumonia (duration was approximately two weeks), event 3 occurred two weeks after event 2 (duration was approximately one week; pegcetacoplan dose was increased), and event 4 occurred two weeks after event 3.

Online Resource Table 2: Treatment-related treatment-emergent adverse events by system organ class and preferred term for PALOMINO (safety set)

| **PALOMINO**  (Phase 2a) | |
| --- | --- |
| **System organ class**  Preferred term | **Pegcetacoplan (270 mg)**  (N=4)  n (%) M |
| **Participants with ≥ 1 TEAE** | 2 (50.0) 52 |
| **General disorders and administration site conditions** | 2 (50.0) 15 |
| Administration site swelling | 1 (25.0) 1 |
| Injection site discolorations | 1 (25.0) 1 |
| Injection site erythema | 1 (25.0) 8 |
| Injection site pruritus | 1 (25.0) 1 |
| Injection site swelling | 1 (25.0) 4 |
| **Nervous system disorders** | 1 (25.0) 2 |
| Dizziness | 1 (25.0) 2 |
| **Skin and subcutaneous tissue disorders** | 1 (25.0) 36 |
| Erythema | 1 (25.0) 36 |

*Abbreviations: TEAE: treatment-emergent adverse event; M: number of events; N: overall cohort size; n: number of patients*

Anti-drug Antibodies (ADA)

A total of 177 serum samples collected during the PADDOCK study were tested for anti-pegcetacoplan peptide antibodies, and only two samples (1.1%, n=2/177; baseline serum samples from two distinct patients) had a confirmed positive result. The observed positive result for anti-pegcetacoplan peptide antibodies in these subjects at baseline had no apparent effect on the efficacy or safety of pegcetacoplan reported for these two subjects. Thirty-five serum samples from the PALOMINO study were screened for anti-pegcetacoplan peptide antibodies and 0% of these samples had a confirmed positive result. Of note, the methodology for the detection of anti-pegcetacoplan peptide antibodies is limited; thus, these data may contain false negative results. Antibodies reactive against the polyethylene glycol portion of pegcetacoplan (anti-PEG antibodies) were detected in a greater proportion of the tested serum samples (PADDOCK, 40.7%, n=72/177 samples; PALOMINO, 5.4%, n=2/37 samples) which may have been related to pre-existing antibodies due to prior polyethylene glycol exposure. Treatment-emergent anti-PEG responses were considered transient during both studies.

Online Resource Table 3: Thrombosis history for PADDOCK and PALOMINO subjects

| Thrombotic events  n (%) M | **PADDOCK**  (Phase 1b) | | | **PALOMINO**  (Phase 2a) |
| --- | --- | --- | --- | --- |
|  | **Cohort 1**  N=3  (180 mg) | **Cohort 2**  N=20  (270 mg) | **Combined Overall**  N=22 | N=4  (270 mg) |
| Subjects with ≥1 thrombosis event in history | 0 | 2 (10.0) 3 | 2 (9.1) 3 | 3 (75.0) 8 |
| Subjects with thrombosis events throughout study protocol | 0 | 0 | 0 | 0 |
| *Abbreviations: TEAE: treatment-emergent adverse event; M: number of events; N: overall cohort size; n: number of patients* | | | | |

Online Resource Table 4: Additional endpoints for PADDOCK and PALOMINO

|  | **PADDOCK Cohort 2** | | **PALOMINO** | |
| --- | --- | --- | --- | --- |
|  | **Baseline (n=20)** | **Day 365 (n=17)** | **Baseline (n=4)** | **Day 365 (n=4)** |
| **Haptoglobin**, mean (SD), g/L  *[NR: 0.14-2.58]* | 0.04 (0.01) | 0.11 (0.12) | 0.10 (0.00) | 0.18 (0.15) |
| **Number of transfusions** in 12 months prior to screening, mean (SD) | 5.3 (4.71) | - | 4.5 (2.38) | - |
| **Transfusion free**,* n (%) | - | 13 (65.0) | - | 4 (100.0) |
| **C3 complement**, mean (SD) g/L | 0.91 (0.20) | 3.42 (0.70) | 1.10 (0.14) | 3.98 (0.28) |
| **CH50 complement**,^†^ mean (SD) | 589.0 (91.75) | 569.0 (72.38) | 58.13 (18.32) | 48.79 (14.40) |
| **AP50 complement**,^§^ mean (SD) | 0.84 (0.27) | 0.46 (0.41) | 2.96 (0.81) | 2.15 (0.71) |
| **PNH granulocytes**, mean (SD) % FLAER | 89.25 (12.97) | 92.90 (6.64) | 71.45 (9.01) | 60.91 (35.05) |
| **PNH monocytes**, mean (SD) % FLAER | 93.33 (6.49) | 93.71 (6.06) | 93.63 (5.00) | 93.55 (4.85) |
| **Clonal distribution of type II and type III PNH RBCs**, % mean (SD) | 39.81 (21.39) | 84.04 (21.04) n=16 | 42.2 (8.06) | 93.0 (6.25) |
| **C3 deposition on type II and type III PNH RBCs**, % mean (SD) | 1.51 (1.80) | 0.44 (0.57)  n=16 | 4.3 (6.10) | 0.1 (0.09) |
| *^*^Subjects were considered transfusion free if they did not have a transfusion during the study pegcetacoplan dosing period.*  ^†^ *Different assays used for the analysis of CH50 between PADDOCK and PALOMINO studies. For PADDOCK, CH50 reported as an activity unit (U); for PALOMINO, CH50 reported as a concentration unit (U Eq/mL).*  ^§^ *AP50 reported without a unit since a normalized value against control was used.*  *Abbreviations: FLAER: fluorescein-labelled proaerolysin; NR: normal range; PNH: Paroxysmal nocturnal hemoglobinuria; RBCs: red blood cells; SD: standard deviation* | | | | |

Online Resource Figure 1: Mean serum levels of pegcetacoplan during the PADDOCK and PALOMINO trials

Smaller dashes on the x-axis indicate additional time points investigated (Day 2, 3, 22, 36, and 43), which were left off the x-axis to not overcrowd the axis. n’s for both trials are listed immediately above the x-axis.


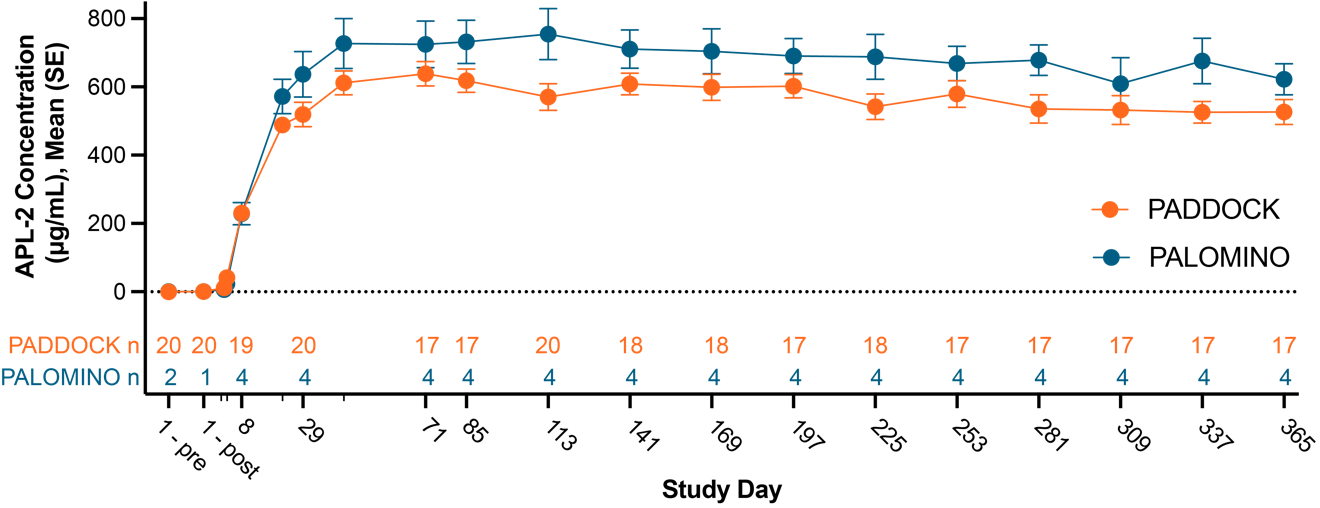


*Footnote: Mean serum levels were measured twice on Day 1; before the first dose pegcetacoplan (Day 1 – Pre) and 1 hour post the first dose of pegcetacoplan (Day 1 – Post).*

*Abbreviations: APL-2: pegcetacoplan; SE: standard error*
